# Neurophysiological and Behavioral Responses to Music Therapy in Vegetative and Minimally Conscious States

**DOI:** 10.3389/fnhum.2013.00884

**Published:** 2013-12-25

**Authors:** Julian O’Kelly, L. James, R. Palaniappan, J. Taborin, J. Fachner, W. L. Magee

**Affiliations:** ^1^Research Department, Royal Hospital for Neuro-disability, London, UK; ^2^Department of Communication and Psychology, Aalborg University, Aalborg, Denmark; ^3^Faculty of Science and Engineering, Wolverhampton University, Wolverhampton, UK; ^4^Department of Neuroscience, King’s College London, London, UK; ^5^Department of Music and Performing Arts, Anglia Ruskin University, Cambridge, UK; ^6^Boyer College of Music and Dance, Temple University Philadelphia, Philadelphia, PA, USA

**Keywords:** EEG, music therapy, disorders of consciousness, assessment, diagnosis, brain injury, vegetative state, minimally conscious state

## Abstract

Assessment of awareness for those with disorders of consciousness is a challenging undertaking, due to the complex presentation of the population. Debate surrounds whether behavioral assessments provide greatest accuracy in diagnosis compared to neuro-imaging methods, and despite developments in both, misdiagnosis rates remain high. Music therapy may be effective in the assessment and rehabilitation with this population due to effects of musical stimuli on arousal, attention, and emotion, irrespective of verbal or motor deficits. However, an evidence base is lacking as to which procedures are most effective. To address this, a neurophysiological and behavioral study was undertaken comparing electroencephalogram (EEG), heart rate variability, respiration, and behavioral responses of 20 healthy subjects with 21 individuals in vegetative or minimally conscious states (VS or MCS). Subjects were presented with live preferred music and improvised music entrained to respiration (procedures typically used in music therapy), recordings of disliked music, white noise, and silence. ANOVA tests indicated a range of significant responses (*p* ≤ 0.05) across healthy subjects corresponding to arousal and attention in response to preferred music including concurrent increases in respiration rate with globally enhanced EEG power spectra responses (*p* = 0.05–0.0001) across frequency bandwidths. Whilst physiological responses were heterogeneous across patient cohorts, significant *post hoc* EEG amplitude increases for stimuli associated with preferred music were found for frontal midline theta in six VS and four MCS subjects, and frontal alpha in three VS and four MCS subjects (*p* = 0.05–0.0001). Furthermore, behavioral data showed a significantly increased blink rate for preferred music (*p* = 0.029) within the VS cohort. Two VS cases are presented with concurrent changes (*p* ≤ 0.05) across measures indicative of discriminatory responses to both music therapy procedures. A third MCS case study is presented highlighting how more sensitive selective attention may distinguish MCS from VS. The findings suggest that further investigation is warranted to explore the use of music therapy for prognostic indicators, and its potential to support neuroplasticity in rehabilitation programs.

## Introduction

The purpose of this paper is to report on a study of neurophysiological and behavioral responses to contrasting auditory stimuli including musical stimuli that underpin music therapy practice in those with Disorders of Consciousness (DOC). Findings will be discussed in relation to our knowledge of these stimuli, and the implications for the development of evidence based interventions in music therapy to address behavioral goals that are important in DOC rehabilitation. DOC comprise a continuum of acquired conditions with two primary diagnostic categories: vegetative state (VS), where there are no discernible indications of consciousness despite evidence of wakefulness (American Congress of Rehabilitation Medicine, 1995) and minimally conscious state (MCS), a condition which may follow VS where consciousness is limited (Giacino et al., 2002). The use of the prefix “persistent” or “permanent” with VS is not currently advocated, as this depiction of the condition suggests irreversibility. Instead, a description of the cause and length of time is recommended (i.e., traumatic VS for 4 months) (Giacino et al., 1997). “Unresponsive Wakefulness Syndrome” has been proposed as a more descriptive and neutral term for VS (Gosseries et al., 2011) although the term has yet to gain widespread usage in the literature. Although coma, where wakefulness and awareness are absent, is sometimes considered as a DOC, this paper will focus on VS and MCS.

Accurately distinguishing between VS and MCS is crucial for decisions regarding treatment, prognosis, resource allocation, and medico-legal judgments (Andrews et al., 1996; Giacino et al., 2002). However, the assessment of people with DOC is a challenging clinical process, highlighted by the fact that the 37–43% recorded misdiagnosis rates in specialist units have failed to change in the last 20 years (Hirschberg and Giacino, 2011). Assessment is confounded by the somewhat arbitrary boundaries between conditions, how judgments regarding the presence of consciousness are based on indirect evidence, and the dynamic, shifting nature of the transition from unconsciousness to consciousness, amongst other factors (Katz and Giacino, 2004). These issues may help to explain why there is little robust epidemiological data on DOC; for example, Beaumont and Kenealy (2005) provide a pragmatic suggestion of the incidence of VS lasting over 6 months lies between 5 and 25 per million of the population for the UK, and the incidence of MCS is yet to be established.

Until the 1990s, clinical consensus was that VS comprised a brain state with intact hypothalamic and brainstem autonomic functions but without capacity for cortical cognitive processes (Monti, 2012). This monolithic conception may be disregarded in the wake of PET and fMRI studies reporting cases of preserved auditory (Laureys et al., 2000), emotional, verbal (Schiff et al., 2002), pain (Kassubek et al., 2003), and language processing (Coleman et al., 2007). Views on the clinical significance of these findings tend to err on the side of caution, suggesting this processing often suffers a disconnect between primary cortex, thalamus multi-modal or limbic regions, and higher order integrative/associative cortices in correctly diagnosed VS (Laureys et al., 2000, 2002; Boly et al., 2004). However, given the challenges of assessment, it is unsurprising that novel passive and active functional imaging paradigm studies report a sub-group of patients diagnosed as VS with intact higher level processing. For example, Monti et al. (2010) highlight the case of an individual able to display evidence of imagining playing tennis and walking around her house, through activation of the supplementary motor area similar to healthy levels. Other studies illustrate similar evidence of volition and awareness in patients erroneously diagnosed as VS (for reviews, see Laureys and Schiff, 2012; Celesia, 2013). Aside from the existence of miss-diagnosed patients, the heterogeneity of DOC has led Bruno et al. (2011) to re-define the “gray areas” between DOC categories, proposing the new categories of “MCS+” where behaviors such as command following and verbal and gestural yes/no responses exist, and “MCS−” where less sophisticated responses stimuli exist, such as visual pursuit or contingent behaviors to emotional stimuli, e.g., smiling when presented with appropriate stimuli.

### Music as a therapeutic intervention with DOC

The rationale for using music therapy to support assessment of DOC may be found in a range of sources. Music is a universal and powerful social medium across cultures and throughout the life span (Blacking, 1976), capable of conveying saliency and emotion, irrespective of verbal content or the need for verbal processing. As such it may provide the optimal stimuli in a field where cognition is severely compromised and stimuli with personal meaning produce greatest behavioral change (Boly et al., 2004; Perrin et al., 2006; Machado et al., 2007). The auditory modality has been established as particularly sensitive to identifying responses indicative of awareness using language based stimuli (Monti et al., 2010), mixed language and non-language based stimuli (Gill-Thwaites, 1997; Gill-Thwaites and Munday, 2004), and musical stimuli (O’Kelly and Magee, 2013b). Differences in the effectiveness of contrasting auditory stimuli in eliciting awareness responses have not been established; however O’Kelly and Magee (2013b) highlight how musical stimuli may be more effective than basic auditory stimuli such as wood blocks, as found in commonly used assessment tools.

The literature on music therapy with DOC comprises divergent ontological approaches, which may be characterized as “music centered/humanist” (e.g., Aldridge et al., 1990; Gustorff, 2002), and “behavioral/pragmatic” (e.g., Baker and Tamplin, 2006; Magee et al., 2013). Despite the divergence in these approaches, there exists a shared belief in the utility of music’s non-verbal and emotional qualities for this work (O’Kelly and Magee, 2013a). To promote arousal and behavioral responses indicative of awareness, procedures using simple improvised melodies entrained to respiration, and live performance of preferred music are advocated (Aldridge et al., 1990; Gustorff, 2002; Magee, 2005; Baker and Tamplin, 2006), together with the systematic assessment of responses to different musical elements (i.e., high and low frequencies) (Magee, 2005, 2007; Daveson et al., 2007). The Music Therapy Assessment Tool for Awareness in Disorders of Consciousness (MATADOC) has recently been standardized to provide reliable data on patients’ responses to a range of musical stimuli (Magee et al., 2013). Another method, “Musical Sensory Orienting Training” or “MSOT” has been protocolized with the goal of stimulating arousal and orientation to time and place (Thaut, 2005). Published research into its efficacy is lacking, however, and overall there exist only a handful of studies reliant on behavioural measures to support the efficacy of music therapy in this field generally (Boyle and Greer, 1984; Boyle, 1994; Formisano et al., 2001). Furthermore, the effectiveness of any one procedure over another in improving important behaviors such as arousal and attention has not been explored. Comparisons of procedures that use different types of music (i.e., salient familiar versus non-salient improvised) would provide guidance for developing optimal and systematic interventions. Greater dialog with neuroscience would also provide mutually beneficial advances in our understanding of how music might be effective in this field (O’Kelly and Magee, 2013a), a factor instrumental in the design of this research. Before detailing the study, it would be useful to summarize the most salient findings of research using the measures adopted herein.

### Physiological measures

Whilst the literature on autonomic nervous system (ANS) activity and emotion is noted for its incongruities and lack of rigor (Kreibig, 2010), there is consensus that heart rate (HR) and its variability (HRV) represent activation and suppression of the sympathetic and parasympathetic nervous system, or arousal, and the body’s homeostatic break on arousal (for reviews, see Berntson et al., 1997; Sztajzel, 2004). HRV may be analyzed in terms of the time domain measures of long or short term nature (SDNN or RMSSD)^1^, or in the frequency domain (LF, HF, ULF, LF/HF)^2^. Time domain HRV is known to decrease during stress or mental work load, with attenuation related to depression (Musselman et al., 1998; Stein et al., 2000); conversely when elevated it has been associated with positive valence found in relaxation (Cacioppo et al., 2000). In relation to music listening, Krumhansl (1997) found more ambiguous results, with increases during sad, fearful, and happy music. In the frequency domain, the LF component is considered correlated with both parasympathetic and sympathetic activity and the HF with parasympathetic activity (Berntson et al., 1997). HR and LF responses are noted as increasing for music listening and performance with reciprocal decreases in HF (Nakahara et al., 2009). Whilst some studies report faster, more rhythmic, music may provide an entrainment effect increasing HR and respiration rate (Bernardi et al., 2006; Gomez and Danuser, 2007), this is not a universal finding (Salimpoor et al., 2009; Dousty et al., 2011). Of particular relevance to this study Riganello et al. (2010) found comparable autonomic changes for both healthy controls and VS patients in normalized LF or “nuLF” induced by complex symphonic music rated as of emotional relevance by controls. Furthermore Wijnen et al. (2006) found in relation to multi-modal stimuli, decreases in parasympathetic activity and increases in sympathetic activity were found to parallel the recovery of consciousness in brain injured patients.

Distinct respiration rates have been linked to different psychological states, such as “fast and deep” for excitement and irregular breathing during emotional upset or task involvement (Boiten et al., 1994). Brown (1962) also found respiration rates faster in those who were actively listening to someone speaking when at rest. Musical auditory stimuli associated with mood states also invoke particular respiration changes, with “happy” music noted as having the most significant effect on respiration (i.e., increasing speed) (Krumhansl, 1997), and respiration increases also noted during musical “chills” associated with pleasurable responses to music (Blood and Zatorre, 2001). Thus respiration rate may provide a simple benchmark for assessing normal psychophysiological responses indicative of preference or positive valence to music. Its utility in the assessment of awareness with DOC has hitherto not been explored in detail in the music therapy literature.

### Electroencephalogram measures

Electroencephalogram (EEG) recording may identify levels of cortical activity corresponding to interoceptive and exteroceptive behaviors and a range of cognitive, emotional, and motor activity, with millisecond accuracy. Amplitude of oscillations provides an indication of the magnitude of active neurons and their synchrony, or numbers of excitatory post-synaptic potentials arriving at a neural assembly at any time point (Varela et al., 2001). As such, EEG offers a non-invasive method appropriate for naturalistic recording of cortical activity in relation to musical stimuli, in real time. Selectively distributed synchronous oscillatory activity within the bandwidths of Delta (δ) frequency at 0.5–3.5 Hz, Theta (θ) at 4–8 Hz, and Alpha (α) at 8–13 Hz, are considered to provide “resonant communication networks through large populations of neurons” (Basar et al., 1999). δ activity is considered important for attention, salience detection, reward behavior (Knyazev, 2012), and internal processing in mental tasks (Harmony et al., 1996). Frontal and frontal midline θ (FMT) are explored extensively in the literature, with putative positive correlations to working and episodic memory, mental effort, sensory motor integration, emotional and internal attention processing, meditative and positive emotional states and, a negative correlation with anxiety (Aftanas and Golocheikine, 2001; Caplan et al., 2003; Ekstrom et al., 2005; Sammler et al., 2007; Mitchell et al., 2008; Fachner et al., 2013). Good cognitive and memory performance is associated with tonic increases in α with reciprocal decreases in θ but with an inverse relationship in relation to phasic or event related activity (for a review of this relationship, see Klimesch, 1999). Both θ and α are considered as core to conscious functioning, through the facilitation of simultaneously different dimensions of cortical integration, and “top down” processing required for such processes as episodic memory retrieval (von Stein and Sarnthein, 2000; Klimesch et al., 2001). Beta (β) activity at 13–30 Hz is associated with focused attention, and cognitive activity, especially in frontal regions (Fernandez et al., 1995). Ratios of θ:β in frontal and other regions are considered to correlate positively with internal attention/relaxation and approach behaviors and negatively with perceived mental effort and anxiety (Howells et al., 2010; Putman et al., 2010; Prinsloo et al., 2013).

As would be expected, differences in DOC EEG behavior compared to controls have been noted; specifically a generalized slowing and dominance of slow wave activity with significantly diminished α power (Kulkarni et al., 2007), and peak power in the θ range in MCS (Schiff et al., 2014). However, attitudes as to the prognostic value of EEG are divided. Some pessimistic accounts suggest the heterogeneity of EEG behavior in this population is too confounding for prognostic value (Kulkarni et al., 2007; Kaplan and Bauer, 2011). Alternatively, Lechinger et al. (2013) has found resting EEG patterns (specifically spectral peaks and ratios of frequencies 8 Hz+ to <8 Hz) to correlate well with behavioral diagnostic assessments. Furthermore event related potentials have been used to assess for recovery (Faran et al., 2006; Wijnen et al., 2007), cognitive processing and cortical learning in VS patients (Kotchoubey et al., 2006, 2009; Kotchoubey, 2007), and have revealed evidence of exogenous and endogenous attention in the absence of behavioral signs of awareness (Chennu et al., 2013). A recent audit of DOC registry data from five rehabilitation centers (*n*: 38 patients) recorded EEG reactivity to acoustic, touch, and light pain stimuli in 54% of patients at initial assessment where prior assessments indicated 21% emerging from MCS, 12% MCS and 67% in VS, although the prognostic value of these measures was not reported (Grill et al., 2013).

### Behavioral measures with DOC

A number of research studies using behavioral measures to determine responsiveness to auditory stimuli in DOC have drawn promising, if unconfirmed, conclusions. The behavioral characteristics of VS occurring in relation to the presentation of multi-modal stimuli have been noted to be heterogeneous, including spontaneous body movement, blinking, and vocalization (Wilson et al., 1996a). Furthermore, contingency between spontaneous body movement and environmental stimuli have been correlated with recovery (Wilson et al., 1996b). Music therapy intervention showed improvements in agitation and interactive behaviors in DOC using blinded behavioral assessment of video behavioral data (Formisano et al., 2001).

From reviewing the literature, it is clear that a range of neurophysiological and behavioral assessment methods are available to investigate the utility of music therapy procedures with DOC assessment, and potential for their use in rehabilitation. The following sections detail a study which addresses the lack of robust research in this area by comparing responses to musical stimuli commonly used in music therapy interventions with other auditory stimuli that provided control and contrasting stimuli.

## Materials and Methods

A multiple baseline within subjects study was used to compare EEG, HR, HRV, respiration, and behavioral responses contingent to music therapy and other auditory stimuli. Ethical approval was given by an internal research review board and a governmental central ethics agency. 20 healthy controls were recruited firstly (13 female aged 24–52 years, mean 34 years, SD 12.5, and 7 healthy males aged 29–59, mean 41, SD 11). Exclusion criteria comprised individuals with known hearing impairment or a high level of musical proficiency. Patients were then recruited who were medically stable, had no known major hearing impairment according to their medical notes, and who were undergoing assessment for diagnosis of awareness using SMART (Gill-Thwaites, 1997; Gill-Thwaites and Munday, 2004) and MATADOC (Magee et al., 2013) assessments. Twelve patients were diagnosed as VS and nine as MCS using SMART with an 86% agreement to MATADOC outcomes, and patients were seen a mean of 7.3 (SD 2.8) months post injury (see Table 1 for demographic and diagnostic details). Control subjects were recruited from staff via email at a large neuro-rehabilitation unit, and patients at the unit via contact with next of kin who provided consultee approval.

**Table 1:** **Patient demographic details and diagnostic outcomes**.

Gender	Age	SMART diagnosis	MATADOC diagnosis	Months since injury	Etiology
Male	49	VS	VS	10	Hypoxic brain injury
Male	23	VS	VS	12.2	Traumatic brain injury
Male	42	VS	VS	3.7	Traumatic brain injury
Male	42	VS	VS	2.2	Hypoxic brain injury
Male	23	VS	VS	14	Traumatic brain injury
Male	22	VS	VS	6.3	Hypoxic brain injury
Male	65	VS	VS	8.2	Hypoxic brain injury
Female	59	VS	VS	6.5	Hypoxic brain injury
Female	54	VS	VS	10.1	Hypoxic brain injury
Female	24	VS	VS/MCS	7.2	Traumatic brain injury
Female	34	VS	VS	9.7	Traumatic brain injury
Female	69	VS	VS	4	Intracerebral hemorrhage
Male	62	MCS	VS/MCS	7.7	Hypoxic brain injury
Male	47	MCS	MCS	4.3	Intracerebral hemorrhage
Male	61	MCS	VS	5.7	Hypoxic brain injury
Male	76	MCS	MCS	6.3	Traumatic brain injury
Male	55	MCS	MCS	8.6	Traumatic brain injury
Female	29	MCS	MCS	5.4	Traumatic brain injury
Female	28	MCS	MCS	9.2	Traumatic brain injury
Female	23	MCS	MCS	5.7	Traumatic brain injury
Female	58	MCS	MCS	6.7	Traumatic brain injury

Five minutes baseline silence (BLS) was followed by the presentation of four contrasting auditory stimuli, with two minutes washout silence separating each stimuli. Music therapy stimuli comprised live performance of preferred song music (LM) and live music featuring simple improvised vocal melody, incorporating the repeated phrase “Hello (*patient’s name*). Hello to music” with a basic supporting accompaniment entrained to respiration (EI). Non-music therapy stimuli comprised digital recordings of disliked music (DM) and white noise (WN). DM was included to provide any evidence of nociceptive or discriminatory responses indicative of awareness, and WN as a non-musical auditory control. LM and EI are typically used in music therapy interventions. Information about personal music preferences for the LM and DM stimuli were obtained from relatives for patients, and directly from healthy subjects. LM and EI were performed by the lead author using a Yamaha NP31 digital electric piano. To control for order effects, the sequence of stimuli was randomized, with order series placed in opaque sealed envelopes for blinded order selection by an independent observer for each participant. Data were recorded using a XLTEC 50 channel video EEG and neuro-physiological data acquisition system with a piezoelectric respiratory belt, and analyzed using Mathworks MATLAB, SPSS (Ver20), and BrainVision Analyzer 2 (BVA) software. ECG data were collected for HR and HRV via two chest electrodes together with respiration data within the XLTEC system. Volume was maintained within a 50–70 dB range for all stimuli using a Tecpal 331 sound level meter. Healthy subjects were instructed to close their eyes half way into each stimulus presentation to provide both eyes open and closed data.

Behavioral data using video recordings of patient sessions were analyzed by a trained volunteer, who was blinded by removing audio from recordings. Ten second segments were scored for a range of behaviors using a graded system from Wilson et al. (1996a) from “eyes shut and no body movement” to “engaged in activity” (e.g., scratching). Any additional behaviors such as blinking and mouth movement were also documented. Commands from the auditory function scale of the CRS R (Giacino and Kalmar, 2006) were presented after each stimulus to observe for behavioral signs of awareness.

Nineteen channels of EEG data were obtained using a common average montage and 10:20 electrode configuration. Due to the presence of craniotomies amongst 13 patients, free electrode placement was adopted in preference to skull caps. Raw data was sampled at 512 Hz and filtered to a hi/low cut off bandpass filter at 0.5 and 30 Hz. Independent Component Analysis with classic sphering and an infomax algorithm in BVA was used to remove artifacts such as eye blinks, followed by Fast Fourier Transformation (FFT) using a hanning window to produce the frequency spectrum, or amplitude as a function of frequency, for each electrode. Data were segmented to 2 sec units and pooled into 21 different electrode configurations to represent different brain regions (see Table A1 in Appendix for details).

After exporting to SPSS, one way repeated measures analysis of variance (ANOVA) analysis with Bonferroni corrections was applied to data. For healthy subjects all data was pooled to provide indicators of healthy responses across measures using ANOVA’s around means. *Post hoc F* statistics were obtained using simple contrasts in relation to BLS to indicate the strength of association of positive or negative change for individual stimuli in contributing to overall ANOVA significance and *F* statistic levels^3^.

Given the clinical and neuro-pathological heterogeneity of those with DOC, particularly with regard to EEG responses (Kulkarni et al., 2007), within-subject statistical analyses were conducted using segmented data that produced individual ANOVAs for the case material.

## Results

### Healthy control data: EEG

**Figure 1:**
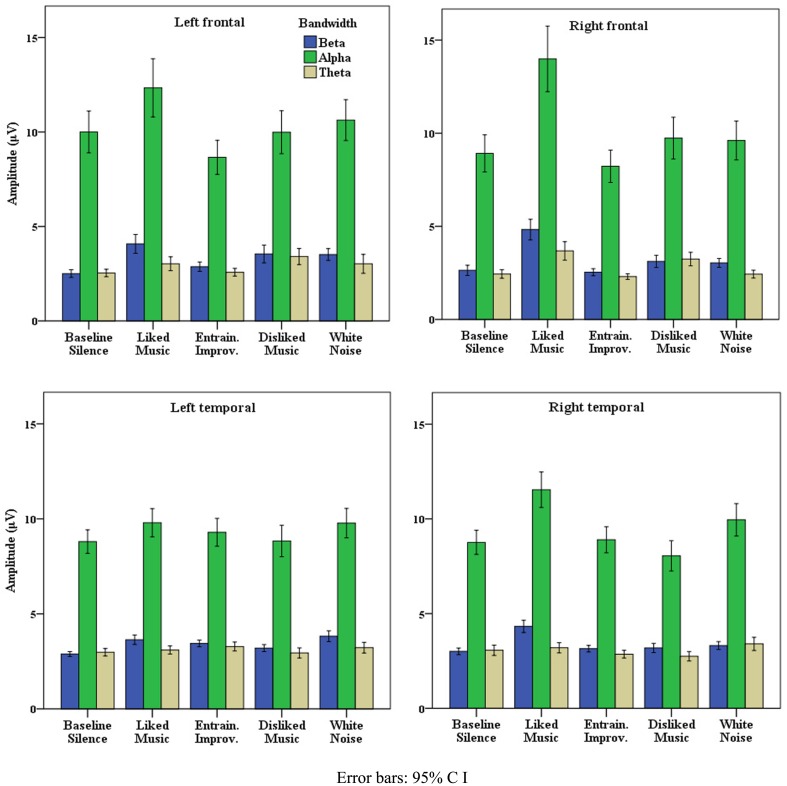
**Healthy frontal and temporal EEG activity**.

**Figure 2:**
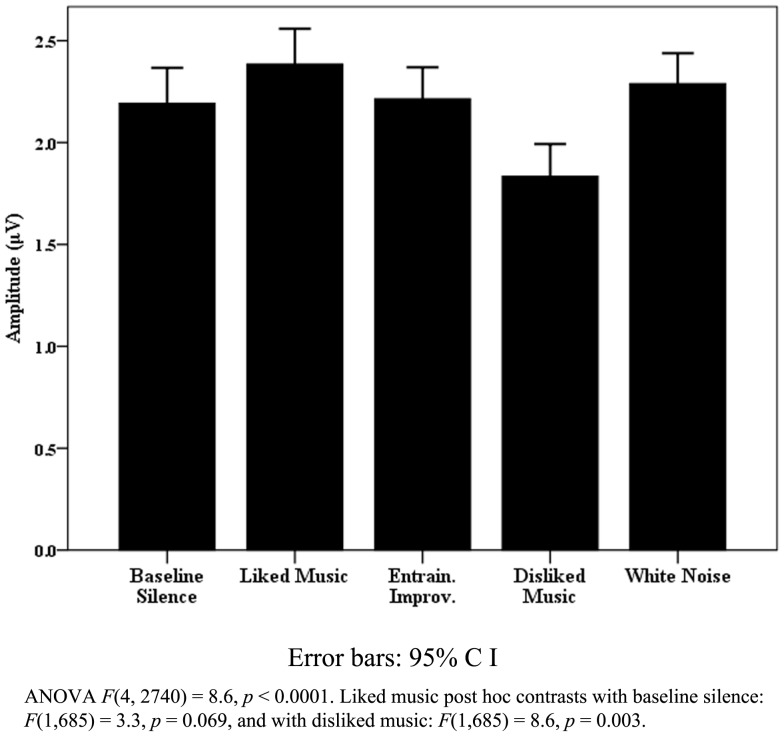
**Healthy frontal midline theta activity**.

Data presented is here for healthy control data from the “eyes closed” sections of recordings, which produced the most artifact free and significant findings across measures, however missing EEG data from one subject resulted from a corrupted signal. Table 2 illustrates that where all electrode data pooled to L and R hemispheres, LM provided the greatest effect on EEG amplitude overall in the R hemisphere, and produced similar increases to WN in the left. The peak for WN in δ over the L and R hemispheres is suggestive of a “drowsiness” effect. Examination of each pooled region revealed the most distinct discriminatory responses occurred in frontal and temporal regions across bandwidths, with a bias toward the right (R) hemisphere generally. Figure 1 illustrates the differences in mean EEG amplitude for frontal and temporal regions, most distinct for LM increases compared to other stimuli in the R frontal and temporal regions, with decreases for EI in α and θ in both frontal regions. Figure 2 highlights significant amplitude differences for θ in the frontal midline region where a contrast between DM and LM was most marked compared to other regions and bandwidths. The ANOVA tests for each pooled region revealed clear significance^4^ within ANOVA’s for changes in δ power in all areas apart from in the left (L) parietal, for θ for all regions except the parietal and R posterior, for α, all regions except the L parietal, and for all individual regions in β frequencies. As the means plots suggest, ANOVA tests revealed right frontal and temporal regions as the most subject to change, most significant for the R frontal region in β [*F*(4, 2740) = 70.7, *p* < 0.001] and α [*F*(4, 2740) = 39.2, *p* < 0.001]. *Post hoc* contrasts with BLS highlighted the dominant contribution of LM to significant ANOVA results in the R frontal region with peak increases in β [*F*(1, 685) = 100, *p* < 0.001] and α [*F*(1, 685) = 50.2, *p* < 0.001].

**Table 2:** **Electroencephalogram mean amplitude (μV): stimuli compared**.

	Left hemisphere					Right hemisphere				
	BL	LM	EI	DM	WN	BL	LM	EI	DM	WN
**BETA**										
Healthy	2.2(1.7)	3.1(3.4)	2.5(2.1)	2.6(2.7)	2.9(2.7)	3.2(3.8)	6.1(12)	4.3(6.3)	4.5(6.1)	3.7(5.2)
MCS	3.4(7.4)	3.1(6.9)	2.6(4.2)	5.4(19)	14.8(73.6)	6.8(30)	2.7(4.6)	4.2(11.7)	3.7(11.9)	11.5(55.4)
VS	1.6(17.1)	2.6(4.4)	1.1(0.7)	1.1(1)	1.9(1.4)	1.5(11)	2.5(2.9)	1.3(1.1)	1.1(0.8)	2(2.2)
**ALPHA**										
Healthy	11.9(16)	11.9(16)	10.4(14.7)	11.4(16)	12(15.7)	11.2(17.7)	13.4(18.9)	10.4(16)	10.3(15.3)	12.4(19.2)
MCS	2(9.8)	2.6(4.2)	1.7(1.6)	1.7(1.6)	2.3(3.3)	1.6(6.4)	2(2.5)	1.7(1.6)	1.5(1.3)	1.9(2.3)
VS	1.3(12.3)	2(2.9)	1(1)	0.9(0.8)	1.7(2.5)	1(7.9)	1.5(1.5)	1(1.1)	0.8(0.7)	1.4(2.4)
**THETA**										
Healthy	2.2(2.3)	2.4(2.7)	2.3(2.4)	2.4(3)	2.41(3.8)	2.3(2.7)	2.7(3.6)	2.1(2.1)	2.4(2.9)	2.3(2.7)
MCS	6.4(5.7)	7(17.8)	7(12.2)	8(9.5)	17.5(27)	6(9.5)	6(11)	7.3(10.1)	10.6(18)	18(29.4)
VS	4.9(23.6)	5.2(8.4)	4.9(6.6)	4.4(6.4)	6(9.6)	4(15.5)	4(15.5)	4.7(7.3)	3.5(4.9)	5(8.6)
**DELTA**										
Healthy	2.9(3)	3.6(4.4)	3.1(4.4)	2.9(3.1)	3.7(4.4)	2.6(2.4)	3.7(5.2)	3.2(3.7)	3(3.4)	3.7(6.1)
MCS	32.6(54)	81(550)	42(198)	45(193)	62.6(890)	47.7(381)	53.5(286)	43(258)	63(493)	62.2(887)
VS	35.2(144)	34.2(89)	43.3(72.5)	43(71.6)	48.8(85)	27.2(127)	29.2(73.7)	40(277)	25.2(41.5)	34.4(55.6)

*SD >100 rounded to 0 decimal point*.

*BL, baseline silence; LM, liked music; EI, entrained improvisation; DM, disliked music; WN, white noise (standard deviation in parentheses)*.

In summary, for the healthy cohort, EEG responses to LM showed dominance in peak power responses globally and across bandwidths compared to the other stimuli tested, particularly in R frontal and temporal regions.

### Healthy physiological data

**Figure 3:**
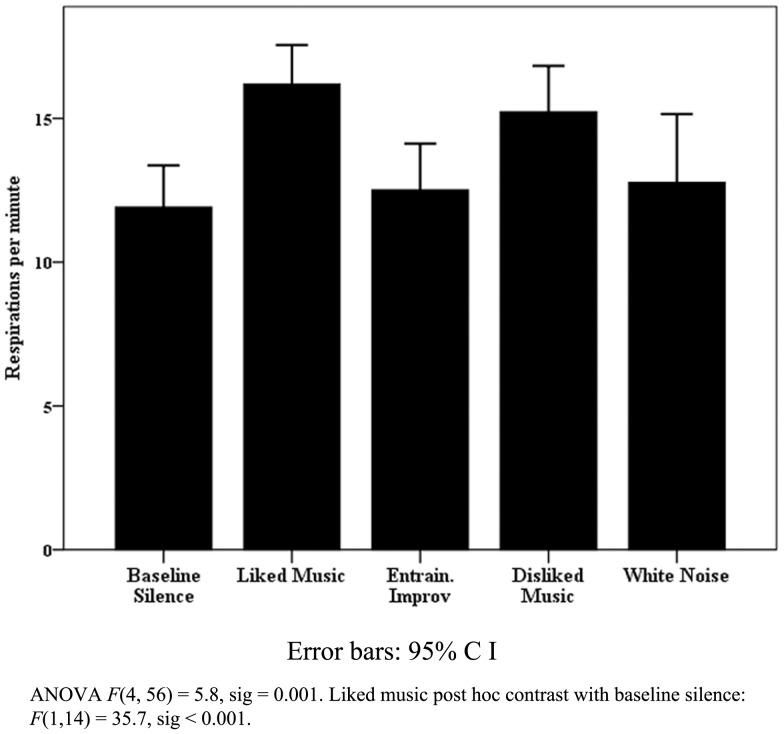
**Healthy respiration rates**.

The most significant findings for pooled healthy data were found for respiration rate and variance. Figure 3 illustrates the significant change found for respiration rate, where LM provided a peak increase^5^ with a mean respirations per minute rate of 17.8 (SD 3.7) compared to a BLS rate of 12.5 (SD 3.2). A significant peak to peak variance was also observed [*F*(4, 56) = 4.1, *p* = 0.006] where WN provided the largest increase [*F*(1, 14) = 11.5, *p* = 0.005]. *Post hoc* correlation analysis of pooled mean respirations per minute compared to means of beats per minute (BPM) for DM, LM, and EI did not indicate entrainment effects upon tonic respiration rate. Significance for HR and HRV was less clearly defined in relation to the effects of individual stimuli. Similarly, in relation to HRV measures, the “eyes closed” LF measure approached significance [*F*(4, 48) = 2.5, *p* = 0.054], but change was non-specific across stimuli, with similar increases in LM, DM, and WN in relation to BLS.

### Patient data

**Figure 4:**
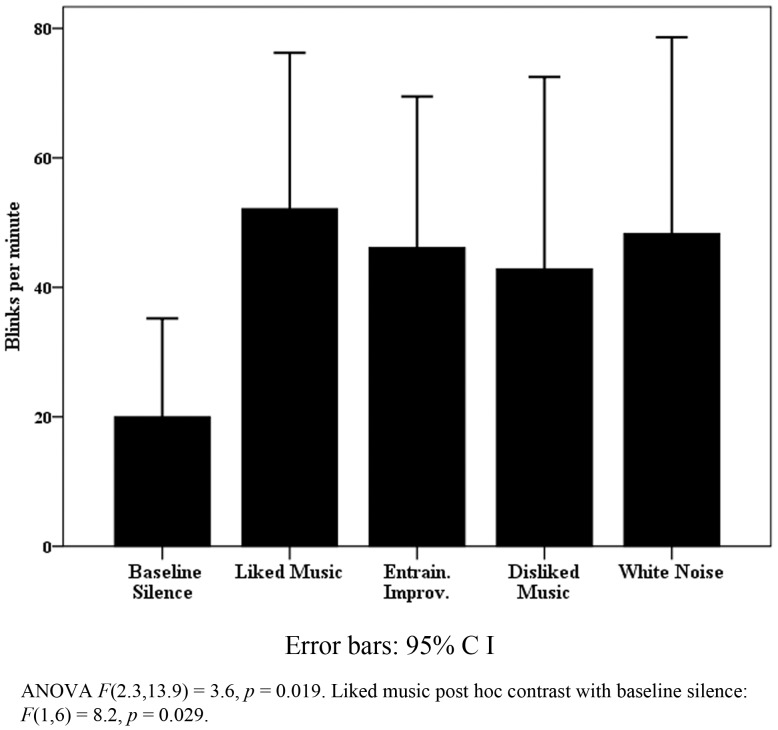
**Vegetative state blink rate**.

All patient data were pooled for observation of trends in patient responses. The results were heterogeneous as expected, particularly for physiological measures, however notable exceptions were found within behavioral and EEG data. Figure 4 highlights that within the VS cohort, pooled eye blink data reached significance, with a peak increase for LM. Similar non-significant trends were observed for LM in eye and mouth movement and “eyes open no body movement” measures in the VS data. Although blink rate change was not significant for the MCS cohort, WN provided a contrasting peak increase [WN mean blinks per min 41.6 (SD 27.1) compared to BLS 34.8 (SD 25.7) and LM 24.3 (SD 25.7)]. Whilst heterogeneous responses and large SD’s contributed to a lack of significant change within the VS cohort generally (see Table 2), mean FMT increased significantly for LM in half (*n*: 6) of cases where ANOVA’s were significant, and peaked significantly in 4 MCS cases (44%).

**Figure 5:**
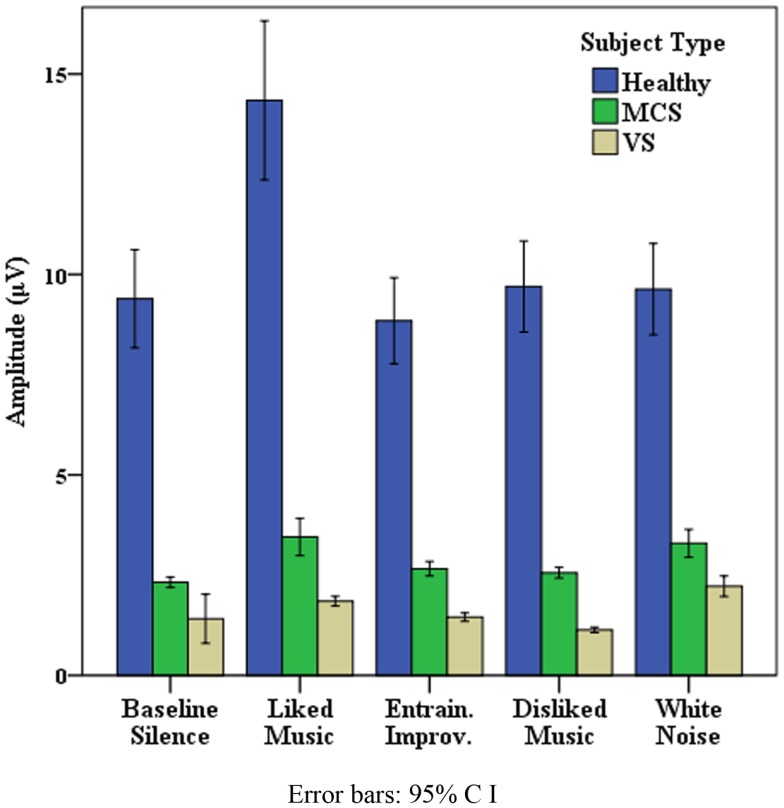
**Frontal alpha activity across cohorts**.

The data provided in Table 2 accords with the literature (e.g., Kulkarni et al., 2007; Schiff et al., 2014) with regard to the typical characteristics of MCS, with a spectral peak in the θ range and VS, where power is predominantly observed in very low frequencies. However, one may also observe LM produced L and R hemisphere amplitude peaks across the MCS cohort in α. In reviewing ANOVA’s for each pooled region, the most noticeable significant change was observed for pooled MCS frontal α [*F*(4, 1850.1) = 36.5, *p* < 0.001] with a peak for LM [*post hoc* contrast *F*(1, 809) = 50.6, *p* < 0.001]. Figure 5 details the frontal α data across cohorts, illustrating expected power differentials between healthy, MCS and VS. An interesting pattern of LM increases for all subjects is also visible, with more distinction from WN increases in MCS than VS subjects. Frontal α peaked for LM in three VS and four MCS subjects, where AVOVAs were significant between *p* = 0.05 and 0.0001. To summarize, whilst pooled physiological patient data were heterogeneous, significant changes were found in relation to an increased blink rate, maximal for LM, across the VS cohort and frontal α for LM across the MCS cohort, with significant FMT increases for LM for half the VS and four MCS patients.

As noted previously, given the general heterogeneity of the patient data, three case studies follow where noteworthy combinations of behavioral and neuro-physiological data are highlighted.

### Case study one

“A” was a 35-year-old female, admitted to the unit with severe TBI following a road traffic accident 10 months previously, who had undergone bi-lateral frontal craniotomies to reduce cranial swelling. Both her SMART and MATADOC assessments gave a diagnosis of VS. Medication adjustments, spiking temperatures, and general poor arousal levels were thought to be impacting on her response levels. The experimental session took place during this assessment period, i.e., when arousal was reduced.

**Figure 6:**
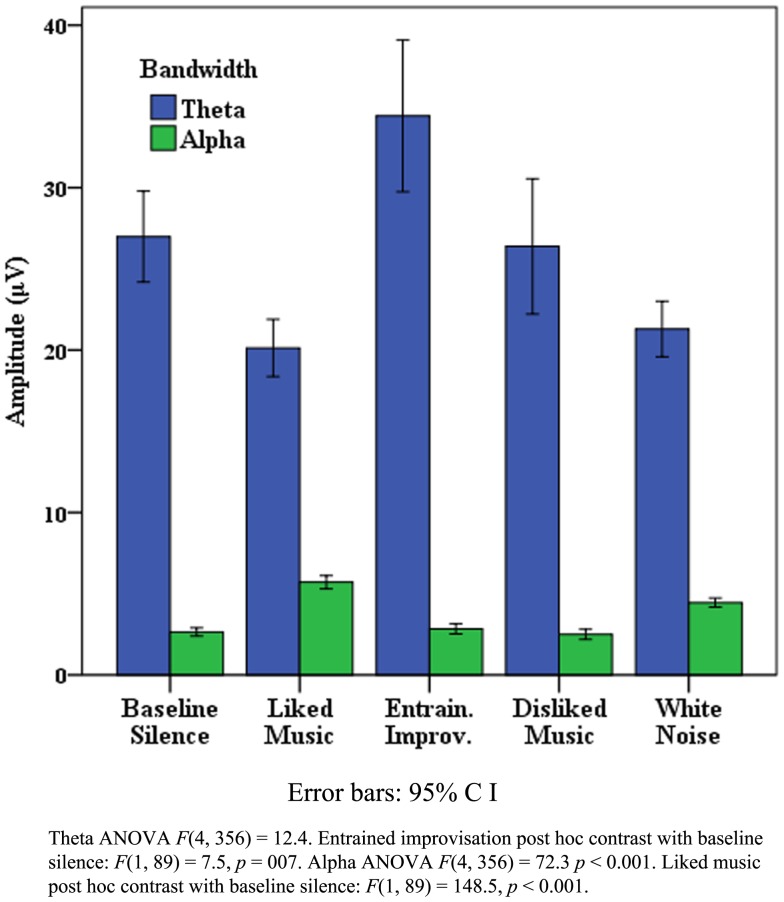
**Case “A” right temporal theta and alpha activity**.

**Figure 7:**
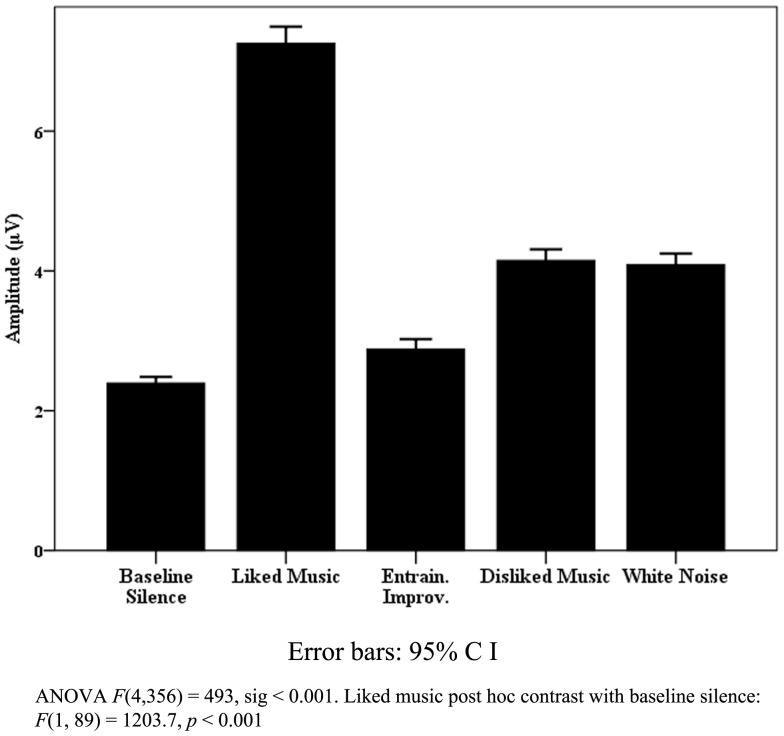
**Case “A” frontal beta activity**.

“A” displayed a range of behaviors suggestive of discriminatory responses in relation to LM, EI, and WN. In relation to EEG, significant *post hoc* peaks for LM and EI were observed for temporal, frontal, central, parietal, and occipital regions across bandwidths. Figure 6 illustrates the θ and α EEG responses for each stimuli in the R temporal region, highlighting the dominance of EI in θ, and LM in α. Furthermore Figure 7 highlights the marked significant increase in frontal β for LM, which contributed to a significant θ:β ratio [*F*(4, 356) = 47.0, *p* < 0.001] with a marked decrease for LM [*F*(1, 89) = 133.8, *p* < 0.001].

“A” had her eyes open with no body movement throughout the session. Interestingly, she displayed eye movement only for LM and WN with significantly more counts for LM (48 compared to 38, *t*-test *p* = 0.001). Her HR ANOVA showed marked significant change [*F*(4, 36) = 1373, *p* < 0.001] due to the striking increases for mean HR for LM (91 BPM, SD: 2) and WN (86 BPM, SD: 1.2) compared to BLS (68 BMP, SD: 0.69), DM (67 BPM, SD: 1), and EI (65 BPM, SD: 0.9). HRV (RMSSD) also showed significant change [*F*(4, 36) = 101.2, *p* < 0.001] due to decreases for LM [*F*(1, 9) = 141.2, *p* < 0.001] and WN [*F*(1, 9) = 154.4, *p* < 0.001]. LF and HF change was also significant [LF: *F*(4, 36) = 11, *p* = 0.012, HF: *F*(4, 36) = 11.8, *p* < 0.001] accounted for by increases in LF for LM [*F*(1, 9) = 9.8, *p* = 0.012] and WN [*F*(1, 9) = 23.8, *p* = 0.001] and decreases in HF for all stimuli compared to BLS. In summary, “A” displayed significant behavioral responses LM, autonomic responses for LM and WN, and EEG amplitude increases in the R temporal and frontal regions across bandwidths for both music therapy stimuli.

### Case study two

“B” was a 49-year-old male with global hypoxic brain injury following a seizure 10 months previously, with high muscle tone and myoclonus^6^. Both SMART and MATADOC assessments gave a VS diagnosis, where he demonstrated mainly reflexive responses, but also some sensitivity to sound and touch, with increased spasm frequency when over stimulated. The experimental session took place during the assessment period.

B’s EEG responses were characterized by the dominance of WN in producing amplitude peaks across bandwidths and stimuli. However, notable exceptions were found for FMT, where a marked peak for EI was observed [ANOVA *F*(4, 356) = 82, *p* < 0.001, EI *post hoc* contrast *F*(1, 89) = 138.1, *p* < 0.001], with further peak activity for LM in β in the frontal, parietal, and midline regions. A significant θ:β ratio was found for the pooled frontal region [*F*(4, 356) = 5.8, *p* < 0.001] with a significant *post hoc* decrease for EI [*F*(1, 89) = 3.7, *p* = 0.57, *F*: 3.7], and increase for WN [*F*(1, 89) = 5.7, *p* = 0.025].

Whilst “B” had his eyes closed throughout the session, various discreet behavioral responses were observed. For example the change in his blink rate (i.e., blinks per 10 s) approached significance [*F*(4, 15.2) = 2.4, *p* = 0.058], due primarily to increases for LM [*F*(1, 17) = 6.6, *p* = 0.02], and head movement changed significantly [*F*(4, 15.2) = 13.1, *p* < 0.001], due to decreases for EI and DM.

Physiological responses for “B” showed significant change in a range of measures, for example his HR decreased for all stimuli compared to BLS [*F*(4, 36) = 4.6, *p* = 0.004], whilst a significant finding for HRV in the RMSSD measure [*F*(4, 36) = 4.2, *p* = 0.004] comprised a contrasting increase for LM [*F*(1, 9) = 4.2, *p* = 0.074] with a decrease for DM [*F*(1, 9) = 4.2, *p* = 0.03]. To summarize, neurophysiological and behavioral data showed a range of significant change, where despite generalized EEG power increases for WN, localized increases across bandwidths were observed for LM and EI, with increases in HRV for LM.

### Case study three

“C” was a 24-year-old female with a R temporal hematoma following a traumatic brain injury 6 months previously. Her SMART assessment completed 2 weeks previously gave a diagnosis of VS. However it was clear from the evidence of reactive θ^7^ at the start of the experimental session, and some consistent responses to the CRS R commands, that she had emerged from VS to MCS. Interestingly her initial MATADOC assessment undertaken concurrently with the SMART gave a more positive borderline diagnosis.

**Figure 8:**
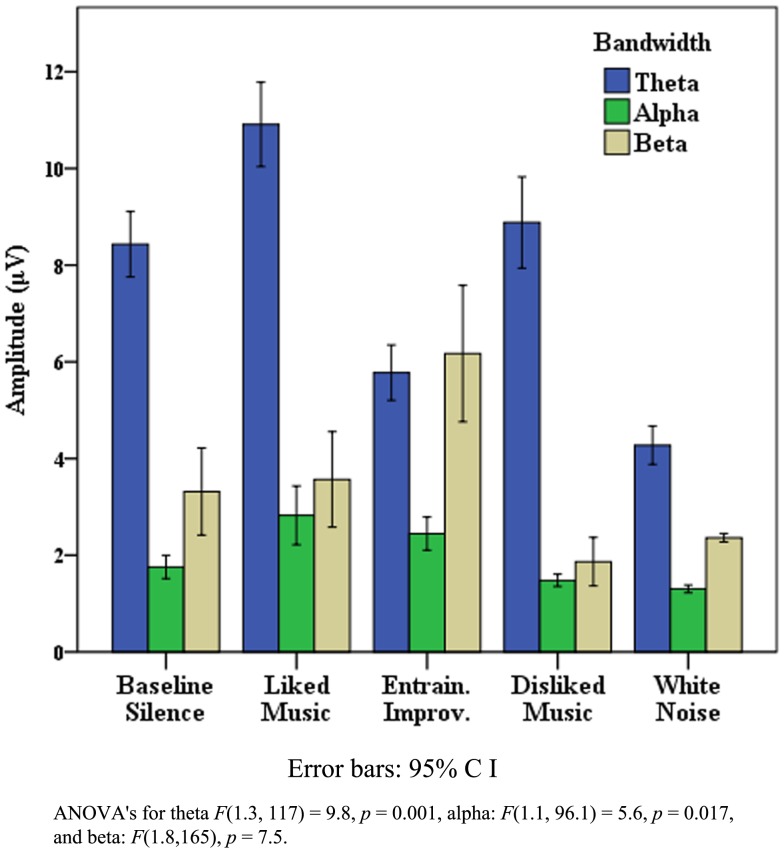
**Case “C”: EEG global responses across bandwidths**.

**Figure 9:**
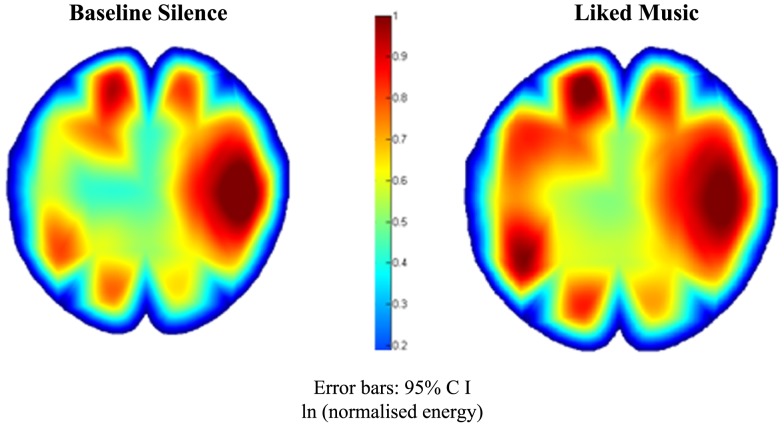
**Case “C” alpha 1 (8–10.5 Hz) EEG topography**.

Figure 8 details global EEG responses for “C” which were notable for the dominance of music therapy stimuli across θ, α, and β, where ANOVAs for each bandwidth regions indicated significant change. In θ, significant increases were found for LM [*F*(1, 88) = 5.4, *p* = 0.022], and decreases for WN [*F*(1, 88) = 24.4, *p* < 0.001]. In α, discrimination was observed in relation to peak power for LM and EI compared to DM and WN, with a shift to EI as the peak stimulus in β [*F*(1, 88) = 14, *p* < 0.001]. θ:β change was significant [*F*(1.2, 96) = 15.6, *p* < 0.00], comprising an increase for LM [*F*(1, 88) = 3.2, *p* = 0.077], with significant decreases for DM, EI, and particularly WN [*F*(1, 88) = 91.7, *p* < 0.001]. Figure 9 highlights the topographic changes observed between BLS and LM energy in low α (8–10.5 Hz), illustrating the relatively less damaged left hemisphere provided the location for increased responses, a pattern repeated across bandwidths for LM and EI.

“C”s behavioral responses were interesting in relation to the finding for blink rate noted previously for VS subjects. Whilst blink rate change was significant [*F*(4, 68) = 3.8, *p* = 0.007], this comprised a decrease for LM [*F*(1, 17) = 3.2, *p* = 0.089] and increase for WN [*F*(1, 17) = 3.1, *p* = 0.096]. However “eyes open body movement” counts also changed significantly [*F*(2.1, 36.8) = 3.9, *p* = 0.026], but conversely here there were no counts for WN in comparison to significant increases from baseline level (*n*: 2) for LM [*n*: 32, *F*(1, 17) = 7.2, *p* = 0.015] and EI [*n*: 28, *F*(1, 17) = 8.35, *p* = 0.15].

“C”s HR changed significantly [*F*(4, 36) = 10.5, *p* = 0.001] due to significant increases for LM [*F*(1, 9) = 41.4, *p* = 0.001], EI [*F*(1, 9) = 17.7, *p* = 0.002], and DM [*F*(1, 9) = 5.1, *p* = 0.05], which was accompanied by significant change for LF [*F*(1, 9) = 7.2, *p* = 0.001], with marked increases for LM [*F*(1, 9) = 46, *p* < 0.001]. HF also changed significantly [*F*(4, 36) = 6.7, *p* < 0.001] accounted for by significant decreases with LM and EI [*F*(1, 9) = 11.9, *p* = 0.007 and *F*(1, 9) = 13.1, *p* = 0.006]. The most contrast in relation to individual measures was within the HRV time domain (RMSSD), where significant change [*F*(1.7, 15.2) = 11.9, *p* = 0.001] comprised a marked decrease for LM [*F*(1, 9) = 55.1, *p* < 0.001] in contrast to an increase for WN [*F*(1, 9) = 6.1, *p* = 0.035]. In summary, the combination of global increases in EEG power across bandwidths for music therapy stimuli combined with the changes noted for behavioral and HRV measures suggests music therapy elicited heightened arousal and mental effort compared to other stimuli. Furthermore “C”s EEG responses indicated heightened discrimination, or selective attention between music therapy and other stimuli.

## Discussion

In parallel with advances in brain imaging techniques such as fMRI, there have been extensive developments in our understanding of how music affects the brain, or “music neuroscience.” However our understanding of the neuro-physiological effects of live music stimuli typically used in therapy interventions is limited (O’Kelly and Magee, 2013a). Whilst some scanning techniques are antithetical to naturalistic explorations of the methods described in this paper, we have adopted EEG and physiological monitoring to provide new perspectives on music therapy with both healthy individuals and those with DOC.

The findings of the healthy cohort EEG analysis of the study revealed widespread increases in EEG power across bandwidths in relation to LM, more distinct in the R hemisphere. This hemispheric specialization accords with Tervaniemi and Hugdahl’s (2003) review comparing speech with music processing, and Stewart et al.’s (2006) review of disorders in music listening. Interestingly, in relation to the literature on valence, this study’s findings are slightly ambiguous. Altenmüller et al. (2002) suggests that greater R frontal activation, as observed here for LM, denotes negative valence in musical listening, particularly in females. This finding may perhaps be accounted for the range of functions preferred music listening serves, such as “self-reflection” (Schäfer et al., 2012). However increased FMT was observed with LM, which is linked to “pleasant” music listening experiences (Sammler et al., 2007). EI, designed to be appropriate for accommodating processing deficits in clinical work, produced a decreases in α compared to silence in the healthy cohort. One may hypothesize that EI lacks the richness and complexity of LM, which may engage more emotional or cognitive responses with healthy subjects. Subjective feedback could have clarified these issues, however this was not gathered as it lay outside the main focus of the study. Valence issues aside, the LM data provides further support to a growing evidence base for preferred music listening and musical activity as beneficial for supporting neuroplasticity through engaging a global system of temporal, frontal, parietal, cerebellar, and limbic brain areas involved in auditory and language processing, emotion, attention, motor control, and memory (Altenmüller et al., 2012; Särkämö et al., 2013). These qualities have been explored with neuro-imaging studies addressing visual neglect, memory, attention, and mood disorders in neurological populations (Särkämö et al., 2008, 2009) and other populations (Koelsch et al., 2010; Fachner et al., 2013), but are as yet untested in this manner in DOC rehabilitation.

The healthy subjects’ respiration data points to a useful benchmark for healthy behavior when the rate increases during listening to preferred music, with a lack of entrainment effect to music tempo suggesting to cortically mediated, emotional, or “top down” processes according to Salimpoor et al. (2009). EEG findings for healthy subjects for DM and WN in the R parietal region combined with WN effects on increasing respiration variance are also noteworthy. Boiten et al.’s (1994) finding that increases in respiration variance are found during emotional upset and Heller’s (1993) model for the R parietal region modulating autonomic and behavioral arousal in “emotional states” suggest both responses correspond to processing emotional responses to auditory/musical stimuli which are likely to be unpleasant.

The findings of the patient component of the study provide a range of support for the use of music therapy in assessment of DOC. Furthermore, they highlight the need for less dichotomous thinking in relation to differences between VS and MCS models, as suggested by Bruno et al. (2011). However, the findings on blink rate with VS, although encouraging, need to be interpreted cautiously in relation to the literature. Whilst blink rate is correlated positively with dopaminergic system activity, arousal (Karson et al., 1990), attention (Abe et al., 2011; Irwin, 2011), and creativity (Chermahini and Hommel, 2010), it has also been *negatively* correlated with attention (reading versus resting state) (Bentivoglio et al., 1997) and recognition of saliency in video stimuli (Shultz et al., 2011). Blink rate increased significantly for VS patients presented with LM, however change was not significant for MCS, although WN provided a contrasting peak increase. This, together with the findings of MCS case “C” (decreased blink rate for LM compared a significant increases for WN, but significant increases in body movement for LM and EI), suggests two types of blink behavior: (i) a basic arousal response where blink rate increases, and (ii) a more sophisticated attention response where blink rate decreases where awareness of the stimuli increases and visual attention is recruited. Further questions are raised by the mean VS resting levels of blinking [20 (SD 16.4) per minute] compared to MCS [34.8 (SD 28.5)], which contrasts with the finding of Bonfiglio et al. (2005) that reduced spontaneous blink rate characterizes the early stages of conscious recovery.

The case studies highlight how significant change may be observed in ANS measures in response to music therapy. The contrasting direction of change for these measures highlights the unique responsiveness of each DOC patient. Based on healthy studies of HRV, and dependent on an individual’s intact emotional processing, it is plausible that some DOC patients may find LM pleasant and relaxing, whilst others may process the stimuli differently, even to the extent of finding the experience stressful in terms of mental exertion. The divergent, yet significant, changes in θ:β ratios in the case studies further highlight this issue. However, without the benefits of subjective verbal accounts such hypotheses are tenuous. The important issue is perhaps not so much whether a brief exposure to a stimulus produces a reaction akin to a “healthy” stress response, more that significant cortical and ANS activity occurs that might suggest discrimination rather than a reflex response. Following the previously noted findings of Wijnen et al. (2006) and Riganello et al. (2010), patients with these significant ANS responses suggest the need for a comprehensive follow up assessment, as these responses may suggest favorable recovery potential.

Despite the previously noted heterogeneity in DOC EEG data, patterns of discriminatory behavior for FMT and frontal α were observed in VS and MCS subjects. Given the literature on FMT as a marker of visio-spatial navigation and memory activity in the hippocampus (Fell et al., 2003; Ekstrom et al., 2005), and cognitive and emotional functions such as motivation, processing information, and attention found in the anterior cingulate cortex (Devinsky et al., 1995; Wang et al., 2005), this finding is noteworthy. Certainly for VS subjects, one would not expect to find any evidence of heightened responsiveness to the complex stimuli of LM or EI in preference to DM and WN, where the former may contain unique elements involved in emotional, language, or memory processing. The frontal α increases in pooled MCS data, and widespread increases in α, θ, and β such as those illustrated in case “C” also underpin the utility of music therapy in promoting cortical activity, which may enhance local and long distance connectivity for MCS patients. Furthermore, the level of differentiation between LM and EI compared to WN and DM in MCS highlight more intact cognitive processes such as selective attention in MCS compared to VS, where this differentiation was less evident. Research exploring whether, and how, these responses can be harnessed to promote neuroplasticity in DOC rehabilitation is indicated. Furthermore, the different patterns of discrimination for VS and MCS found highlights the potential of combined music therapy/EEG assessment as providing a non-invasive and widely applicable method to compliment behavioral assessments.

The data collection for the patient cohort in particular posed numerous challenges, such as applying EEG electrodes where patients had craniotomies, to removing numerous EEG artifacts caused by appliances such as feeding machines. Patients did not receive auditory brain stem testing to exclude patients with undiagnosed hearing impairment, which may have provided a confounding element to the findings. Furthermore, all patients were receiving a range of medications, where a common side effect was drowsiness, which may compromise EEG and ANS responses. Conversely one may consider the significant change found in neuro-physiological measures in this study as important from a clinical perspective, where behavioral responses alone, attenuated by drowsiness, might give a different impression. The provision of music therapy methods by one music therapist, and sample size, also suggest caution is needed when interpreting findings, particularly in relation to the potential variance in performance style one might find with other music therapists. It should also be noted that LM and DM comprised heterogeneous tempo, harmonic, rhythmic, and lyrical content. This lack of standardization may have provided a confounding effect, perhaps explaining the divergent HR and HRV results. However, it would be antithetical to provide these musical items in a standardized form, as this would possibly obscure any elicitation of responses based on intact memory function. Further research might involve several therapists trained within standardized protocols, and larger samples to improve the robustness of findings.

## Conclusion

This study addresses the lack of empirical evidence supporting music therapy in the assessment of those with DOC, with pilot level data on the neurophysiological and behavioral responses of DOC patients to music therapy. By comparing healthy data with findings from the DOC cohorts it is evident that music therapy is capable of eliciting a range of responses indicative of arousal and selective attention. Combined music therapy and neuro-physiological assessment may provide distinctive contribution revealing intact responsiveness to salient stimuli, even in VS patients considered to be “unaware” of themselves and their environment, which merits further investigation for prognostic value. Furthermore, as some VS subjects responded selectively, and beyond chance level to music therapy in neuro-physiological measures, it is clear that blanket assumptions as to the “unresponsive” nature of VS are questioned, especially where these assumptions are predicated on behavioral assessment alone. Given our expanding knowledge of the role of musical activity in promoting neuroplasticity, further research is needed to explore the potential of music therapy to optimize assessment, and promote functional gains within this population.

## Conflict of Interest Statement

The authors declare that the research was conducted in the absence of any commercial or financial relationships that could be construed as a potential conflict of interest.

## Footnotes

^1^SDNN, standard deviation of all normal to normal peak intervals; RMSSD, root mean square of successive differences between adjacent peak intervals.

^2^LF, low frequency; HF, high frequency; ULF, ultra low frequency; LF/HF ratio, ratio of low-hi frequency power.

^3^*F* statistics are rounded to the first decimal point. ANOVA’s were computed using alpha = 0.05.

^4^Significance hitherto defined by *p* ≤ 0.05.

^5^“Increases” and “decreases” henceforth in relation to *post hoc* ANOVA contrasts with BLS.

^6^A brief, involuntary twitching of a muscle or a group of muscles caused by sudden muscle contractions.

^7^i.e., clear θ wave present in the EEG display, showing spiking responses to sudden noise or brief painful stimuli (e.g., pinching).

## Appendix

**Table A1:** **Brain regions and pooled electrodes**.

Brain region	Electrodes
Left hemisphere	A1, C3, F3, F7, Fp1, O1, P3, T3, T5
Right hemisphere	A2, C4, F4, F8, Fp2, O2, P4, T4, T6
Left frontal	F3, F7, Fp1
Right frontal	F4, F8, Fp2
Frontal	F3, F4, F7, F8, Fp1, Fp2
Left temporal	T3, T5
Right temporal	T4, T6
Temporal	T3, T4, T5, T6
Left parietal	P3
Right parietal	P4
Parietal	P3, P4
Posterior left	O1, P3, T5
Posterior right	O2, P4, T6
Posterior	O1, P3, T5, O2, P4, T6
Occipital	O1, O2
Central left	C3
Central right	C4
Central	C3, C4
Frontal midline	F3, Fz, F4
